# Trace minerals in tilapia fillets: Status in the United States marketplace and selenium supplementation strategy for improving consumer’s health

**DOI:** 10.1371/journal.pone.0217043

**Published:** 2019-06-06

**Authors:** Razieh Farzad, David D. Kuhn, Stephen A. Smith, Sean F. O’Keefe, Nicholas V. C. Ralston, Andrew P. Neilson, Delbert M. Gatlin

**Affiliations:** 1 Department of Food Science and Technology, Virginia Polytechnic Institute and State University, Blacksburg, Virginia, United States of America; 2 Department of Biomedical Sciences and Pathology, Virginia-Maryland College of Veterinary Medicine, Blacksburg, Virginia, United States of America; 3 Department of Earth System Science & Policy, University of North Dakota, Grand Forks, North Dakota, United States of America; 4 Department of Fisheries and Wildlife Sciences, Texas A&M University, College Station, Texas, United States of America; University of Illinois, UNITED STATES

## Abstract

This goal of this study was to highlight the importance of minerals in the diet of fish for meeting micronutrient requirements in the human diet. First arsenic, calcium, cadmium, copper, iron, molybdenum, magnesium, manganese, sodium, phosphorus, potassium, selenium, and zinc concentrations of twelve commercially available tilapia samples were measured. The nutritional value of fillets in regard to their mineral content were assessed to establish potential health benefits or risks for consumers. The health benefit value of selenium was also calculated. Positive health benefit values indicate that tilapia fillets in the United States marketplace of this study do not pose health risks associated with mercury exposures. Selenium was the trace mineral of interest. After the market study, a seven-week fish feeding trial was conducted to study the influence of organic versus inorganic dietary selenium on Nile tilapia (*Oreochromis niloticus*). Fish were fed two different diets enriched with the same concentration (0.01g kg^-1^) of selenium in form of inorganic (sodium selenite) or organic (seleno-L-methionine) selenium in triplicate groups. There were no significant differences between growth and biometrics of fish fed different diets (*p>*0.05). At the end of trial twelve fish from each treatment were collected. Fillets of fish fed organic selenium had selenium concentrations of 0.55 ± 0.01 μg g^-1^ which were significantly (*p<*0.05) higher than fish fed inorganic selenium at levels of 0.22 ± 0.008 μg g^-1^ or fish samples from the marketplace with a selenium level of 0.2 ± 0.03 μg g^-1^. Fish fed organic selenium also had significantly higher (*p<0*.*05*) plasma and kidney selenium in comparison to fish fed inorganic selenium. No significant differences (*p>0*.*05*) were observed in glutathione peroxidase activities in either the plasma or liver of Nile tilapia in the different treatment groups. This study shows that organic selenium is a better option for production of Nile tilapia fillets rich in selenium.

## Introduction

Malnutrition is one of the biggest problems facing the world [1]. Fish is a protein rich food that is increasingly favored by consumers. Being packed with nutrients fish can play an incredible role in fighting the malnutrition problem [2, 3]. Fish are not only rich in high quality protein but are also one of the primary sources of Omega 3, polyunsaturated fatty acid (PUFA). Polyunsaturated fatty acid can decrease cardiovascular disease and is essential for brain development. In addition, fish are good sources of highly bioavailable micronutrients such as minerals and vitamins that play important role in human health, growth and development and disease prevention [4]. However, the important role of fish as dietary sources of micronutrients is poorly recognized and under evaluated [5].

With rising pressures on capture fisheries, global fish demand is increasingly being met from aquaculture with aquaculture producing more than half of the fish for human consumption [6]. As the aquaculture industry is growing it is embracing a nutrition sensitive approach and is moving beyond maximizing productivity to improving nutrition and health of the animal. Production of healthier fish with better nutritional quality can in return improve the health of human consumers. Herein the value of aquacultured product was increased by producing the fillets that are fortified with a mineral which enhance the functionality of already healthy product.

Being part of numerous enzymes, minerals participate in many vital biological processes for human and animals. In this context selenium (Se) is one of the most interesting elements. Selenium is the functional component of selenocysteine (Sec), a Se containing amino acid genetically encoded for incorporation into the active sites of unique proteins (selenoproteins) which are expressed in all vertebrates [7]. One of the best studied selenoproteins is glutathione peroxidase (GPX, EC 1.11.1.9) [8]. This enzyme protects the cell membranes against oxidative damage by catalyzing the reactions that are essential for the conversion of hydrogen peroxide and fatty acid hydroperoxides to water and fatty acid. Glutathione peroxidase is measured to evaluate the bioactive status of Se in humans and animal tissues. Selenium is important for various aspects of human health, including optimal immune response, proper thyroid hormone metabolism, cardiovascular health and prevention of neurodegeneration and cancer and antagonism effect against methylmercury and other heavy metal toxicities[9,10,11,12,13].

Selenium is also very important for maintaining the health of fish. Selenium deficiency in fish can compromise the immunity and increase mortality of the animal. Previous studies have provided evidence that farmed fish may require a higher content of Se in their diet, and if it is delivered in an organic form it may better accumulated in tissue, and be more biologically active [14,15].

Due to the beneficial effects of Se for human and animal health this element has been proposed as a valuable resource for including in functional feeds for aquaculture which can promote health and growth of fish as well as improving the quality of their respective fillets for human consumption. When supplementing feed with Se, an important consideration is which form of Se to use [16,17] Supplementation of feed with organic Se can potentially produce fish with higher concentrations of Se for humans to consume.

Therefore, in this study first a small-scale market survey was conducted to evaluate the mineral and heavy metal composition of tilapia fillets available in the U.S. marketplace. The market study aimed to provide information for consumers on potential contribution of consumption of tilapia fillet to mineral contents of their diets. The concentrations of heavy metals were compared to the available fish mineral composition data, standards, and safety limits to evaluate any health risk associated with consumption of this fish regarding heavy metal toxicity. In addition, Se specific nutritional benefit in relation to mercury (Hg) exposure risk associated with tilapia consumption was assessed by calculating health benefit value of Se (HBV).

After the market study, a fish trial was conducted to evaluate the effect of Se species as functional ingredients in the diet of Nile tilapia, *Oreochromis niloticus*.

The effects of dietary organic and inorganic Se on growth performance, biometrics, plasma and hepatic GPX activity and tissue assimilation of Se was compared to identify species of Se that lead to higher tissue and plasma concentrations. The effect on fish production was evaluated by measuring food conversion ratio (FCR), growth rate, survival, and intestinal microvilli length. The information on Se concentration of tilapia fillets obtained in market study was used to evaluate the success of supplementation of aquafeed with organic and inorganic Se for fortifying Nile tilapia fillets with Se. This was done by comparing the Se concentrations of fillets from market survey to Se concentrations of tilapia fillets from fish trial.

## Materials and methods

### Market study

#### Market samples

Twelve tilapia samples were purchased from various grocery stores in four different regions of the United States. Samples were collected from the mid-Atlantic (Blacksburg, VA), mid-west (Chicago, IL), northeast (Boston, MA and New York City, NY) and southeast regions (Plano, TX). Data regarding type of culture (wild caught versus farmed raised), country of origins, price, product certification and fish condition were recorded upon purchase (Table 1). Samples were freeze-dried and stored at -20 °C in accordance with United States Environmental Protection Agency (USEPA) guidance for fish tissue analysis [18]. Freeze dried samples were microwave digested and mineral concentrations were measured using the methods below.

**Table 1:** Tilapia fillets sampled from various regions of the United States.

Sample	Region	Super market	Price*	Farmed	Storage condition	Boneless	Skinless	Whole	Gutted	Certification^❖^	Origin
**T1**	Mid-Atlantic	Kroger	5.20	Yes	Frozen	Yes	Yes	No	Yes	None	China
**T2**	Mid-Atlantic	Walmart	3.92	Yes	Frozen	Yes	Yes	No	Yes	None	Columbia
**T3**	Mid-Atlantic	Walmart	7.94	Yes	Frozen	Yes	Yes	No	Yes	None	China
**T4**	Mid-Atlantic	Food lion	4.99	Yes	Frozen	Yes	Yes	No	Yes	None	China
**T5**	Midwest	Aldi	5.99	Yes	Frozen	Yes	Yes	No	Yes	Yes	China
**T6**	Midwest	Whole food	7.98	Yes	Fresh	Yes	Yes	No	Yes	None	Ecuador
**T7**	Northeast	Trader joes	5.99	Yes	Frozen	Yes	Yes	No	Yes	None	Thailand
**T8**	Northeast	Stop and shop	6.99	Yes	Frozen	Yes	Yes	No	Yes	Yes	China
**T9**	Southwest	Kroger	5.99	Yes	Frozen	Yes	Yes	No	Yes	None	China
**T10**	Southwest	Market street	5.99	No	Fresh	Yes	Yes	No	Yes	None	Equator
**T11**	Southwest	Market street	6.99	Yes	Fresh	Yes	Yes	No	Yes	None	Mexico
**T12**	Southwest	Tom toms	6.99	Yes	Fresh	Yes	Yes	No	Yes	None	Costa Rica

*Price is per pound of fillet

^❖^Certification is a market-based measure for evaluation of aquaculture and capture fisheries in various objects form food safety, quality, and traceability to environmental and social impact.

#### Reagents and material

Standard solutions for analyzed elements (HPLC grade [S, Ca, Cd, Cu, Fe, Hg, Mb, Mg, Mn, Na, P, Se, and Zn], Sigma Aldrich, St Louis, MO, USA) were used from 1000 mg L^-1^ stock solution to develop calibration curves. All aqueous solutions of reagents and standards were prepared using ultra-pure water (Milli- RO 12 plus Milli-Q purification system for water, Millipore, Bedford, MA, USA). Trace grade (35% w/v) nitric acid (Sigma Aldrich, Sigma Aldrich, St Louis, MO, USA), and hydrogen peroxide (30% w/v) (Sigma Aldrich, St Louis, MO, USA) were used for digestion of the fish fillets digestion. All glass and polythetrafluoroethyelene (PTFE) vessel liners were cleaned by soaking in 20% v/v HNO3 for 24 h, then triple-rinsed with ultra-pure water and dried.

#### Microwave digestion

Samples were digested using a modified method adopted from Olmedo et al, [19]. Briefly, 0.5 g of each sample was weighed into MARS 5 microwave Teflon vessels (CEM Corporation, Matthews, NC, USA) in duplicate, that had been subjected to an anti-static gun. Samples were digested using 8 mL of 35% (w/w) nitric acid and 2 mL of 30% hydrogen peroxide (w/w). Before digestion, the vessels were briefly swirled to mix and allowed to sit open for approximately 10 minutes. The microwave program performed a ramp to 180 °C over 20 minutes and then held at that temperature for 10 minutes before cooling to 50 °C. After digestion, the samples were transferred to polypropylene (PP) tubes and the vessels were quantitatively rinsed using 2% (w/w) nitric acid.

#### Inductively Coupled Plasma Mass Spectroscopy (ICP-MS)

The freeze-dried digested samples were diluted to two separate dilutions based on analyte (i.e. mineral) with 2% (w/w) nitric acid prior to analysis. The samples were diluted approximately 200-fold for the higher concentration metals such as Na, K, P, and Ca. To accommodate trace metals, the samples were diluted 10-fold. The dilutions were necessary to reduce the concentrations of acids going on to the instrument as well as to reduce contamination and to allow the analyte concentrations to fall within the calibration range. The calibration standards and ranges for each element are shown in Table 2. Sample acquisition was performed on an Agilent 7900 ICP-MS (Agilent, Santa Clara, CA, USA) using a custom method that utilized the helium mode octopole reaction system (ORS) to reduce and/or remove potential matrix interferences while simultaneously maintaining the lower detection limits needed for the analysis [20]. A certified reference material (DORM-4, National Research Council of Canada) was digested and analyzed along with the samples to ensure proper digestion and analysis.

**Table 2:** Calibration standards and ranges for each analyzed element.

Metals	Calibration Concentration (ng mL^-1^ solution)					
Na, Mg, P, K, Ca, Fe	10	50	100	500	2,000	5,000
Zn	1	5	10	50	200	500
Mn, Cu, As, Se, Mo, Cd, Pb	0.1	0.5	1	5	20	50

#### Health benefit value of selenium (HBV)

Health benefit value of Se was used to determine Se-specific nutritional benefits in relation to potential Hg exposure risks presented by tilapia fillets available in the U.S. marketplace. The Hg and Se mass concentrations (parts per million) for each individual fish sample were converted to molar concentrations (μmole kg^-1^). To determine whether the amounts of Hg and Se present in the fish fillets would potentially result in a Se insufficiency or a net excess, the difference in the molar concentrations of Se and Hg was divided by Se concentration which gives the absolute molar concentration present in the fish fillets. This results in the relative amount of Se available [21].

RelativeSeavailability=([Se−Hg])/Se

To reflect the physiological amount of Se that is potentially provided or lost in respect to sequestration by Hg, the relative amount of Se available was multiplied by the total amount of Hg and Se that was presented in the fish fillets.

HBV=([se−Hg])/Se×(Se+Hg)

Positive HBV means that the fish has Se in molar excess to Hg and thus are unlikely to pose a health risk [21].

#### Risk and nutritional assessment

The mineral concentration in a 100 g serving of fish fillet was calculated and converted to wet weight basis by considering the moisture content (range of 74 to 81%) of the samples. For nutritional assessment, the calculated intake of the analyzed essential elements per 100 g serving of tilapia fillets were compared to Dietary Reference Intake (Recommended Daily Allowance and Adequate Index) for children, adult females and adult males aged between 14 and 50 years that is set by the Food and Nutrition Board, Institute of Medicine, United States National Academy of Sciences [22–24].

### Fish trial

#### Fish trial experimental design and diet

All procedures have been approved by Virginia Tech’s Institute of Animal Care and Use Committee (VT-IACUC-#16–099). A 7-week grow out trial was conducted to evaluate the effect of dietary supplementation of inorganic versus organic Se on Nile tilapia. One hundred and sixty-eight tilapia were acquired from Genetic Spring (Akvaforsk Genetics Center, Miami, FL, USA). After a month of acclimatization, fish with an average weight of 170 ± 2 g, (Mean ± SE) were arbitrarily stocked into six polyethylene tanks (284 L each) in a single recirculating aquaculture system (RAS) at 28 fish per tank. The RAS system was equipped with bead and sand filters for mechanical filtration, bioreactor for nitrification, UV disinfection units, heat exchangers, and distributed diffuse aeration.

Two separate isonitrogenous and isoenergetic diets (Table 3) [25] were prepared. The dry ingredients were mixed followed by the addition of oil and distilled water. The mixture was pelleted (Mill 72A, Lawson Mills Biomass Pellet Solutions, Ebenezer, PEI, Canada) and then dried at room temperature for 24 h. Experimental diet nutritional proximate and trace mineral concentrations for the various diets are presented in Table 4. Feed was then stored in a commercial refrigerator at a temperature between 0–3.5 °C until it was used. Diets were supplemented with 1 mg kg^-1^ of Se in forms of sodium selenite (2.83 mg kg^-1^) and seleno-L- methionine (2.19 mg kg^-1-^). Concentration of Se in each diet was measured using Inductively Coupled Plasma Mass Spectroscopy (ICP-MS) analysis as described above. Dietary Se concentration were measured to be 2.01 mg kg^-1^ for the inorganic Se diet and 2.23 mg kg^-1^ for organic Se diet.

**Table 3:** Formulation of basal diet (g kg^-1-^as is basis).

Ingredients	Inorganic Se supplemented diet	Organic Se supplemented diet
Soybean meal	526	526
Dextrinized starch	217	217
Menhaden fish meal	117	117
Soy oil	51	51
CaPO4, dibasic	11	11
Glycine	11	11
DL-Methionine	2	2
Vitamin premix	5	5
Mineral premix	1	1
Celufil	37	37
CMC	21	21
Stay-C	1	1
Seleno-L-Methionine	0	0.01
Sodium selenite	0.01	0

**Table 4:** Experimental diet nutritional proximate and trace mineral concentrations.

Proximate level (g kg^-1^)	Inorganic Se supplemented diet	Organic Se supplemented diet
**Crude Protein**	379	381
Carbohydrate*	281	288
**Ash**	86	87
**Total Fat**	69	71
**Crude Fiber**	85	79
**Moisture (%)**	10	9.4
**Trace mineral level (mg kg**^**-1**^**)**		
**Iron**	341	348
**Copper**	14	14
**Zinc**	243	244
**Manganese**	67	74
**Selenium**	2.0	2.2

*Calculated value (Merrill and Watt, 1973): Carbohydrate = total-(ash+ crude protein + moisture+ total fat)

Each diet was fed to fish in triplicate tanks. During the trial fish were weighted weekly on a per tank basis. The amount of feed was adjusted based on the weight gain and feed rates (2–3% of fish body weight) were consistent between the two treatment groups. Feeding rate was on a percent body weight per day basis. Feed were loaded on 24 belt feeders (four per tank) to deliver feed hourly over an 18-hour period. Water quality parameters (Table 5) were analyzed using methods adapted from APHA [26] and HACH [27] as follows: Dissolved oxygen and temperature were measured using a meter (YSI ProDO, Cole-Parmer, IL, USA) daily. Alkalinity was also measured daily using a titration method. Nitrite, nitrate, total ammonia nitrogen and pH were measured three times a week. Approximately half of the sump tank (95 liters) water was exchanged daily to prevent potential Se accumulation in the water. Also, the Se content of water in each tank was measured at the beginning, middle and end of the experiment to check for any Se accumulation in the tanks.

**Table 5:** Mean water quality results for the RAS system during the 7-week trial. Number of sampling events is denoted by n.

Dissolved Oxygen (mg L^-1^) n = 48	Alkalinity (mg L^-1^ as CaCO_3_) n = 48	Temperature (°C) n = 48	Nitrate-N (mg L^-1^), n = 7	Nitrite-N (mg L^-1^), n = 21	Nitrite-N (mg L^-1^), n = 21	pH n = 21	Total ammonia-N (mg L^-1^), n = 21	Se Conc. (μg L^-1^), Day 1 . n = 3	Se Conc. (μg L^-1^), Day 28 n = 3	Se Conc. (μg L^-1^), Day 49 n = 3
5.3 ± 0.2	125.5 ± 5.5	29 ± 0.2	11.0 ± 1.7	0.08 ± 0.03	0.08 ± 0.03	7.78 ±0.05	0.3 ± 0.03	0.45 ± 0.01	0.5 ± 0.04	0.54 ± 0.02

Values are mean ± standard errors

#### Health and production characteristic of fish

Food conversion ratio, survival rates, and weight gain were used to compare the production between the two treatment groups. Survival was assessed daily and FCR was determined by dividing amount of feed provided for consumption by the weight gain of fish on a per tank weekly basis. Weight gain was calculated weekly. Also, the hepatosomatic index (HSI) and viscerosomatic index (VSI) biometrics were assessed for each treatment group at the end of feeding trial.

#### Sampling

At the end of the 7-week feeding trial, 12 fish from each diet were arbitrarily collected. Fish were netted individually and euthanized with sodium bicarbonate buffered tricaine methanesulfonate solution (MS 222, 250 mg/L, Western Chemical, Ferndale, WI, USA). Weight and length of each fish were measured and recorded before necropsy.

#### Plasma collection

Blood was collected postmortem from the caudal vessels of each fish using a 1mL syringe and 23-gauge needle. Blood samples were placed in ethylenediaminetetraacetic acid (EDTA) coated tubes and centrifuged for 15 minutes at 10,000 x *g*. After centrifugation, plasma samples were collected and stored at –80 °C until analyzed. Total Se concentration and GPX activities were measured in individual plasma samples.

#### Fillet collection

After blood collection, each fish was filleted by the same person for consistency in sampling. Fillets were weighed for calculation of fillet yield. Fillet samples for total Se concentration were freeze-dried and stored at –20°C. Fillet yield was determined by dividing the fillet weight by the whole weight of each fish.

#### Liver, kidney and intestine collection

The viscera of individual fish were collected and then the liver and the total viscera weights (g) were measured individually. Liver, kidney and intestine samples were collected for microscopy, analysis of GPX activity and Se content of the tissue. Intestinal samples were preserved in 2.5% glutaraldehyde for transmission electron microscopy (TEM). Liver and kidney samples were collected were stored at –80 °C immediately after collection. Hepatosomatic index and VSI were measured by dividing the liver weight and viscera weight by the whole fish weight respectively.

#### Total GPx activity measurement

Liver and plasma GPX activity were measured using a commercial kit (Cayman Chemical, Ann Arbor, MI, USA). This assay is based on the method that measures the decrease in absorbance at 340 nm (A_340_) while NADPH is oxidizing to NADP^+^. The rate of reduction in A_340_ is directly proportional to GPX activity of the sample [28,29]. Before analysis, liver samples were homogenized in 10 mL of cold buffer (50 mM Tris HCl, pH 7.5, 5 mM EDTA and 1mM Dithiothreitol (DTT) per gram of tissue. Homogenized samples were centrifuged for 15 minutes at 10,000 x *g*. The supernatant was collected, and protein concentrations of samples were measured using a bicinchoninic acid (BCA) assay kit (Fisher Scientific, UK). The BCA assay is based on the method from Lowry et al. [30]. Plasma samples were diluted 1:2 with cold buffer (50 mM Tris-HCl, pH 7.6, containing 5 mM EDTA and 1 mg mL ^-1^ of bovine serum albumin (BSA)) prior to analysis.

#### Transmission electron microscopy

Sections of the intestine from each treatment group were preserved in 2.5% glutaraldehyde buffered with 0.1M sodium cacodylate trihydrate. The samples were washed twice in 0.1M Na sodium cacodylate trihydrate for 15 minutes. Washed samples were post-fixed in 1% OsO4 in 0.1M Na cacodylate for 1 hour, washed again 0.1M Na cacodylate twice for 10 minutes, then dehydrated in graded ethanol solutions for 15 minutes at each stage [31]. Propylene oxide was used to further dehydrate the samples. Dried samples were then infiltrated with a 50:50 solution of propylene oxide: Poly/Bed 812 for 24 hours, thereafter with a 100% mixture of Poly/Bed 812 for 12 hours per manufacturer’s instructions (Polysciences Inc., Warrington, PA, USA). Blocks prepared for TEM were sectioned with a diamond knife and ultramicrotome into 90-150nm sections on 200 mesh grids A JEOL 1400 scope (JEOL Ltd., Akishima, Tokyo, Japan) equipped with a Gatan Orius SC 1000 CCD camera (Gatan Inc. Pleasanton, CA, USA) was used to obtain TEM images. Collected images (magnification x 15000) were analyzed using ImageJ (National Institute of Health, USA) [32] and were used to measure microvilli length.

#### Statistical assessments

Statistical analysis was performed using GraphPad Prism for Mac (La Jolla, CA, USA) and JMP 13(SAS institute, Cary, NC, USA). All data were analyzed using t-test to evaluate significant differences among the means. Significant differences were indicated at *p<0*.*05*.

## Results

### Market study

**Fig 1:**
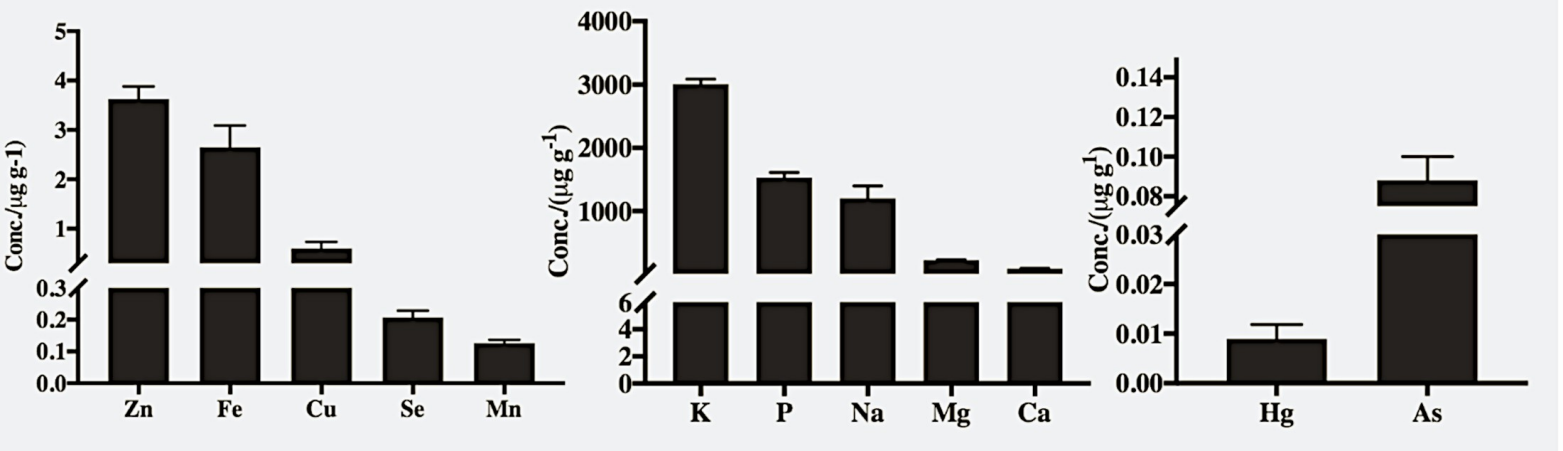
Minerals concentration (*μ*g g^-1^ of wet weight) of tilapia fillets from the United States market place. (a) trace minerals (b) essential minerals (c) heavy metals. Cadmium concentrations were below detection limit in all the tilapia fillets. Data is represented as means ± Standard Errors, n = 12.

Eleven of the 12 tilapia samples available in U.S. marketplace were farmed raised. China was the main country of the origin (Table 1). Good recovery of analyte was achieved with a combination of HNO_3_/H_2_O_2_ (4:1) mixture for digestion of all the samples using microwave-assisted digestion. The recovery values of DORM-4 reference material were between 87–111% for the elements that were being analyzed. Concentrations of essential and trace minerals and soft electrophiles in tilapia fillets are presented in Fig 1. The major microelements were K, P, Na, Mg and Ca, followed by micronutrients Zn, Fe, As, Se, Cu, Mn, and heavy metals Hg, As, and Cd.

#### Health benefit value of selenium

Tilapia fillets had a positive HBV_Se_ of 11.83 ± 4.95.

#### Contribution to Dietary Reference Intake (DRI)

The contribution of tilapia fillets per 100 g serving to mineral’s DRI is summarized in Table 6 for children and adults. Tilapia fillets provide more than 10% of DRI of P, Se and Na for adult, and more than 10% of DRI of Cu, Mg, P, Se and Na per 100 g of serving for children.

**Table 6:** Contribution (%) of tilapia fillets to dietary reference intake (DRI)^❖^ of each element for children and adults by gender.

	Ca	Cu	Fe	Mg	Mn	Mb	P	Se	Zn	K	Na
**Children**	0.93	13.6	2.65	17.57	0.08	1.29	31.5	68.9	7.26	8.08	10.5
**Male**	0.82	6.93	2.94	5.93	0.63	0.66	17.8	39.0	1.45	6.14	12.6
**Female**	0.80	6.93	2.04	7.32	0.63	0.66	17.8	39.0	4.54	6.14	12.6

^❖^Dietary Reference Intake (Recommended Daily Allowance and Adequate Index) for children, adult females and adult males aged between 14 and 50 years is set by the Food and Nutrition Board, Institute of Medicine, United States National Academy of Sciences [22–24].

### Comparison of mineral composition to USDA data

Concentrations of minerals of tilapia fillet of this study was compared with USDA composition data for raw tilapia fillets which is shown in Table 7. Tilapia fillets from market survey had significantly different concentrations of Ca, Fe, Mg, Mn, K, Na, Cu, and Zn compared to USDA composition data.

**Table 7:** Comparison of mineral concentrations (mg/100 g) of fillets from market study to USDA compositional data for tilapia fillets.

Mineral	Market Study Data	USDA Composition Data
Ca	9.2 ± 0.3	14
Fe	0.26 ± 0.04	0.69
Mg	17 ± 0.7	34
P	116 ± 7.8	204
K	303 ± 7.6	380
Na	125 ± 20	56
Zn	0.35 ± 0.2	0.41
Cu	0.059 ± 0.006	0.075
Mn	0.012 ± 0.0004	0.037

Values are mean ± standard errors

### Fish trial

#### Growth performance

Growth performance of fish fed the experimental diets is presented in Table 8. No mortalities were observed during the trial. Fish that were fed organic Se had slight, but insignificantly higher (*p<0*.*05*) growth and lower FCR than fish fed inorganic Se. There were also no significant differences between the FCR, HSI or VSI of fish fed the different diets.

**Table 8:** Production parameters of Nile tilapia, *Oreochromis niloticus*, fed inorganic or organic Se for 7 weeks.

Treatment	Survival (%)	Weight gain (g)	FCR*	Weekly weight gain (g)
Inorganic Se	100%	333±101	1.43±0.15	41.0±6.7
Organic Se	100%	362±110	1.27±0.08	45.0±5.9

*FCR: Food Conversion Ratio

#### Se content in fish tissue and plasma

**Fig 2:**
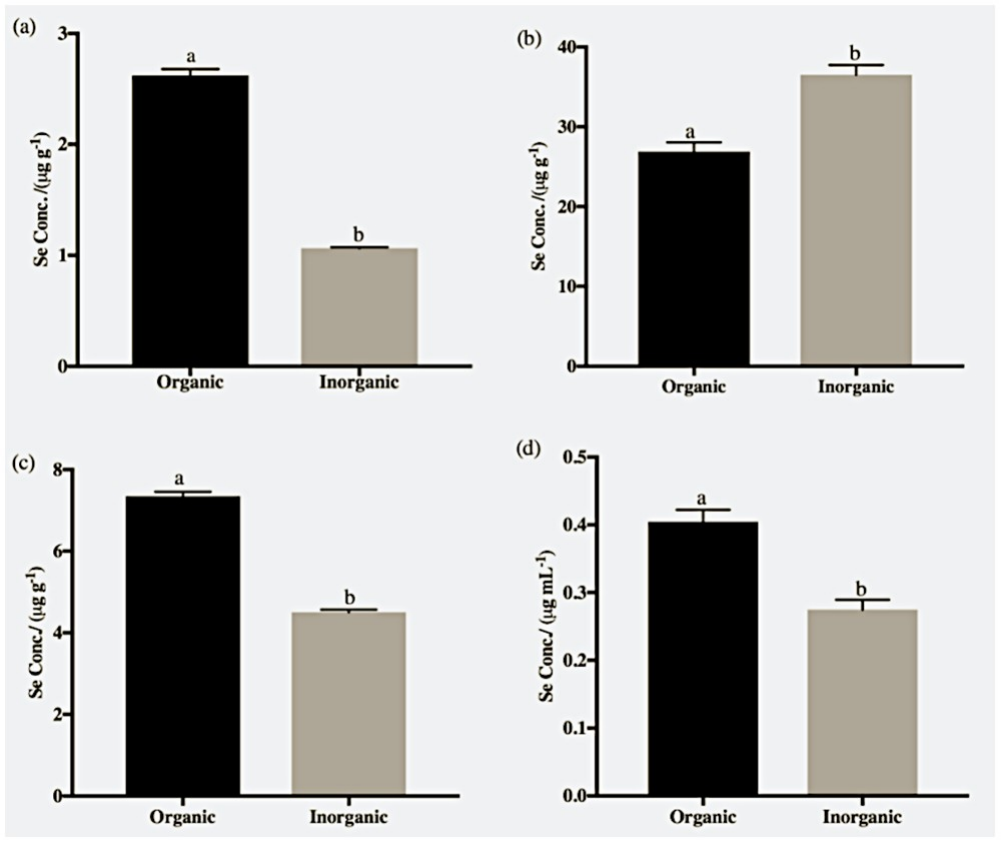
Selenium concertation (*μ*g g^-1^ of dry weight) in (a) fillets, (b) livers, (c) kidneys and (d) plasma of Nile tilapia, *Oreochromis niloticus*, supplemented with seleno-L-methionine (organic) and sodium selenite (inorganic) in basal diet. Means with different letters were significantly different (*p<0*.*05*). Data is represented as mean ± Standard Errors, n = 6.

Selenium concentration was significantly higher (*p<0*.*05*) in plasma, kidney and fillet of fish fed organic Se (Fig 2). However, the concentration of Se was higher in the liver of fish fed inorganic Se. Overall, Se accumulation was higher in the liver and kidney compared to fillets or plasma compared with the fish fed inorganic Se.

#### Total GPX enzyme activity in plasma and liver

**Fig 3:**
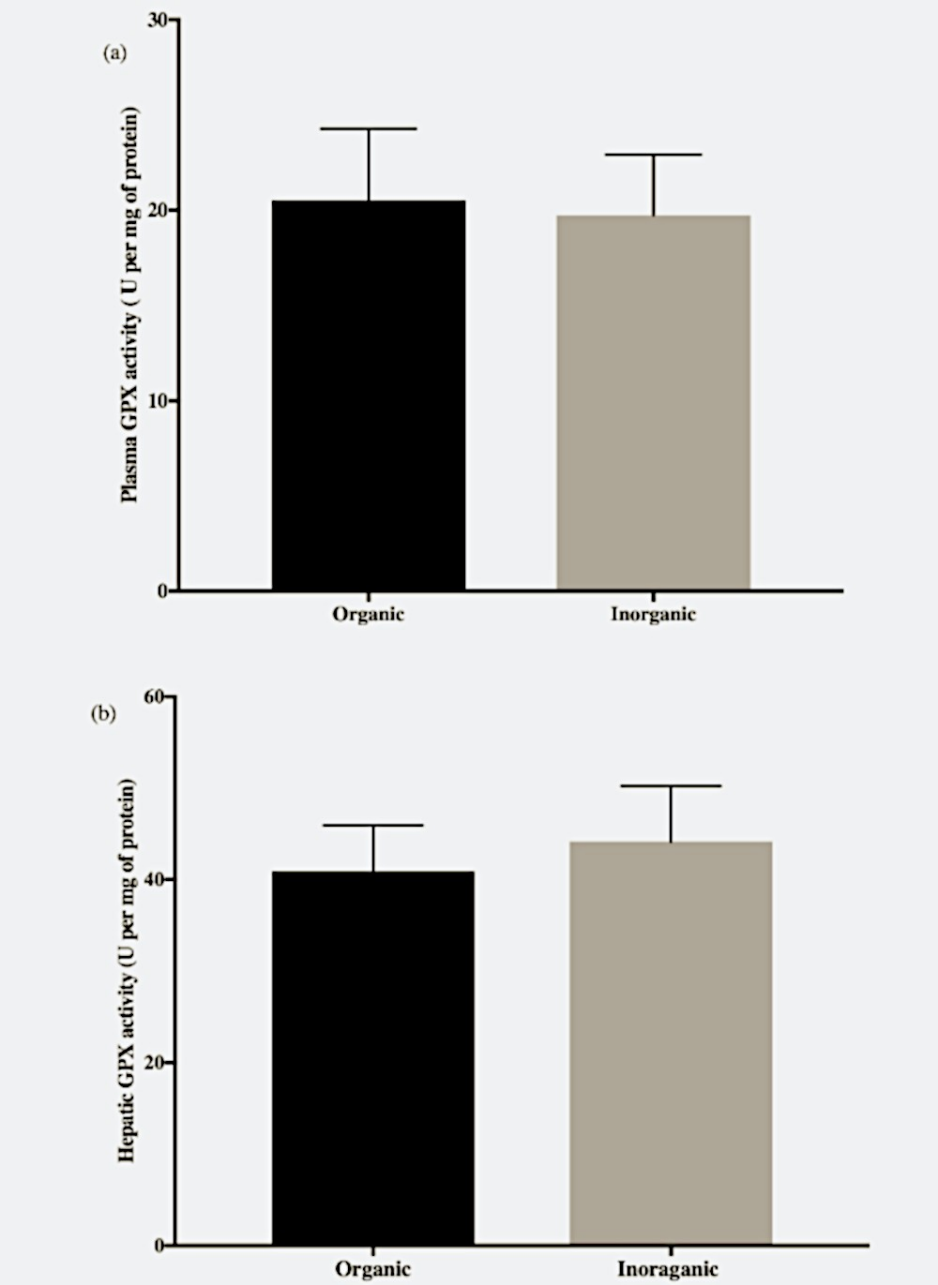
Glutathione peroxidase activity of Nile tilapia, *Oreochromis niloticus*, in (a) plasma and (b) liver supplemented with seleno-L-methionine (organic) and sodium selenite (inorganic) Se in basal diet. Unit (U) is nano moles of NADPH oxidized per milligram of total protein per minute. There were no significant differences (*p>0*.*05*) in hepatic and plasma activity indicating both forms of Se were sufficient to meet tissue need for maximizing GPX activity.

There was no significant difference in total GPX activity in liver or plasma of fish that were fed the different experimental diets (Fig 3).

#### Microvilli size

**Fig 4:**
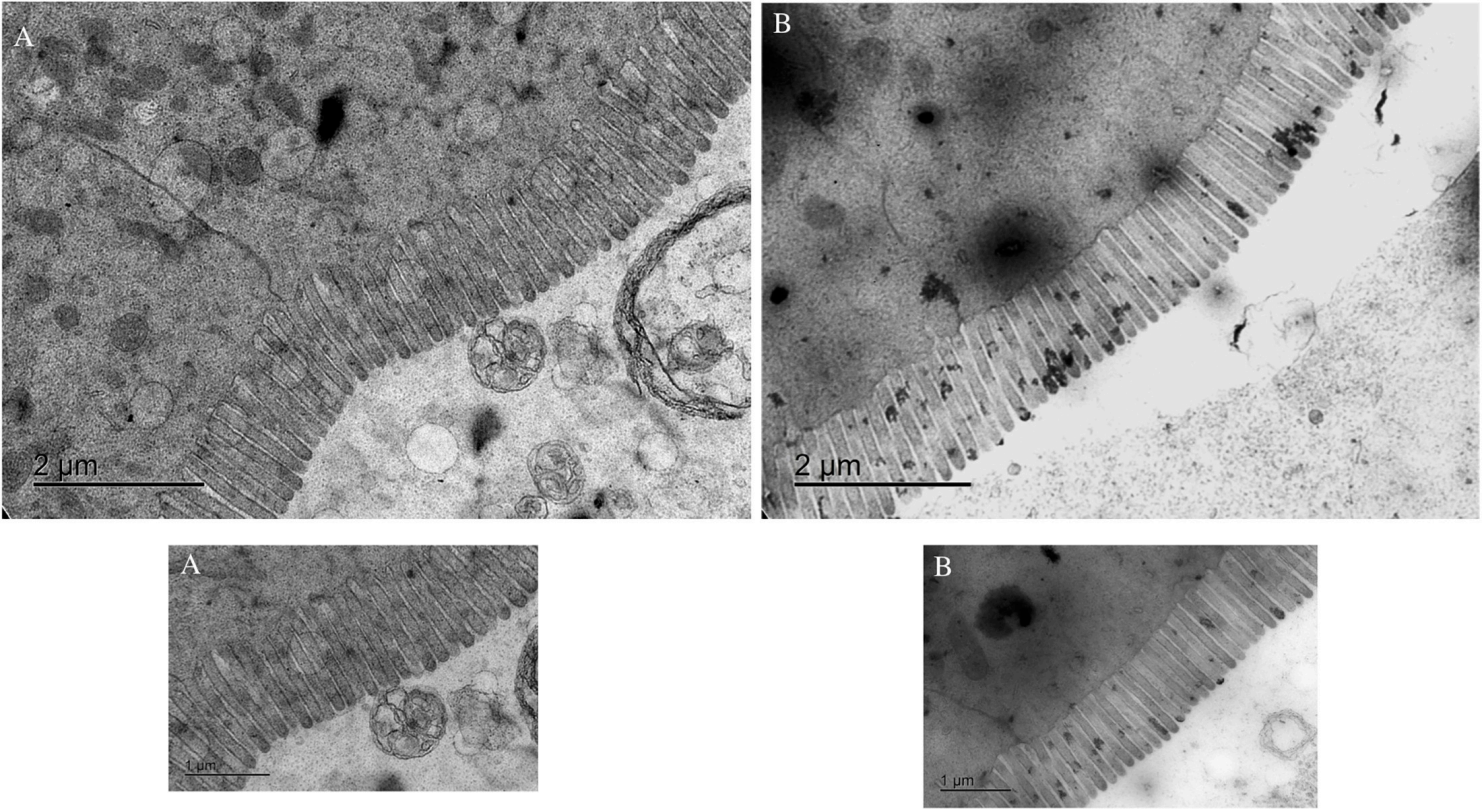
Representative transmission electron microscopy (TEM) images of Nile tilapia, *Oreochromis niloticus*, microvilli fed (a) sodium selenite (inorganic Se) and (b) SeMet (organic Se).

There were no significant differences between the microvilli from inorganic and organic Se fed group (Table 9). Representative TEM images of the fish fed organic and inorganic Se is presented in Fig 4.

**Table 9:** Biometrics of Nile tilapia, *Oreochromis niloticus*, fed inorganic or organic Se for 7 weeks.

Treatment	HSI*	VSI^❖^	Fillet Yield	Microvilli length^✧^(μm)
Inorganic Se	1.0 ± 0.06	6.0 ± 0.2	24.0 ± 1.3	1.2 ± 0.04
Organic Se	1.0 ± 0.07	6.4 ± 0.03	26.0 ± 0.9	1.3 ± 0.02

Values are mean ± standard errors of 12 samples per diets. Fillets were boneless and skinless.

*HSI: Hepatosomaic Index

^❖^VSI: Visoerosomaltic Index

^✧^ Refer to Fig 3 for TEM images of microvilli

#### Comparison of Se content of tilapia fillets from market to tilapia fillets from fish trial

**Fig 5:**
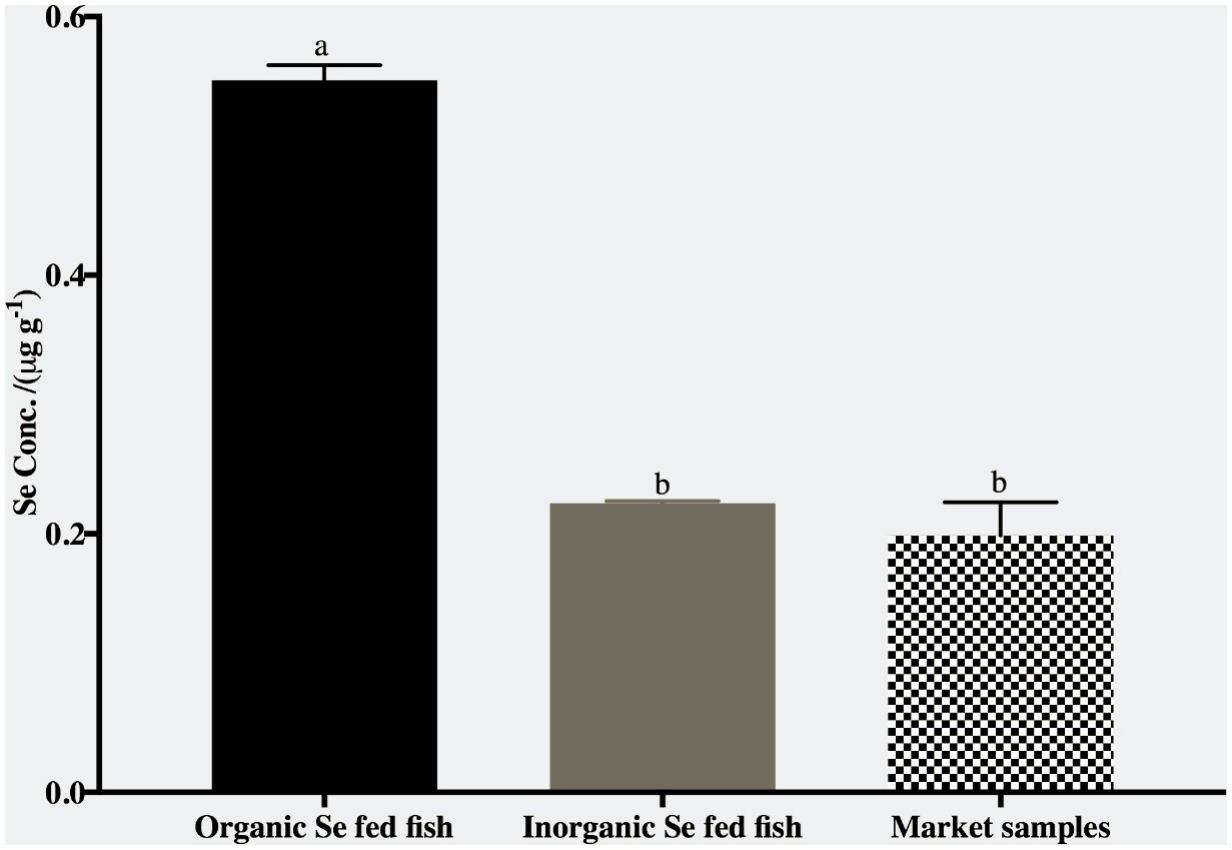
Comparison of Se concentrations (*μ*g g^-1^ of wet weight) of tilapia fillets from U.S. marketplace to tilapia fillets from fish nutritional trial. Means with different letters were significantly different (*p<0*.*05*).

Organic Se fed tilapia had significantly higher (*p<0*.*05*). concertation of Se in comparison to inorganic fed fish or tilapia samples from commercial markets. There were no significant differences between tilapia samples from the market and inorganic fed fish (Fig 5).

## Discussion

Elemental composition of 12 commercially available tilapia samples in U.S. marketplace was analyzed. Among the main elements studied, K had the highest concentration followed by P, Na, Ca, Mg. Previous study has shown that higher concentration of Na than K in the diet can significantly increase the risk of cardiovascular disease [33]. Concentration of Na was lower than K in all the tilapia fillets samples of the present study demonstrating that these products from marketplace are good choice of a meal for a healthy diet. Total concentrations of As, Cd and Hg were measured and compared to food safety standards showing that the studied fish products do not pose health risks associated with heavy metal exposures. Cadmium concentration in all the samples were below detection limits which agrees with previous research that found only trace amounts of Cd in tissues of aquatic organisms [34]. In nature, As exists in both organic (i.e. arsenobetaine and arsenocholine) and inorganic forms, however in fish tissue it is present mostly in organic forms which are less toxic than the inorganic species [35]. Tilapia fillet total As (organic and inorganic) concentration was significantly lower(*p>0*.*05*). than USEPA limit of 1.3 mg kg^-1^ (wet weight) in freshwater fish for human health protection [36]. Mercury is one of the most toxic heavy metals in the environment. Methylmercury (MeHg) is a contaminant in fish that can pose risks to humans. High MeHg exposures can have severely adverse effects on the central nervous system, particularly the developing brain of a fetus [37]. However, instead of harm, epidemiological studies have consistently found that increasing Hg exposures from maternal consumption of ocean fish during pregnancy result in substantial maternal health benefits and improved neurological outcomes in their children [38–43]. Clearly, it is not the increased Hg exposures that were responsible for these positive health outcomes. Instead, the improved neurological development, motor abilities, verbal intelligence, perception, social behavior, and diminished hyperactivity appear to be due to improved maternal intakes of nutrients such as omega-3 fatty acids and Se which are abundant in ocean fish [44].

Mercury concentrations in tilapia fillets analyzed in the market survey were similar to the U.S. FDA report on Hg concentrations of fish and shellfish [45]. The U.S. FDA action level for Hg is 1.0 mg kg^-1^(ppm wet weight), but many countries have level of 0.5 mg kg^-1^ as acceptable Hg concentration in fish tissue [46,47]. Mercury concentration in all the tilapia fillets in this study were under 0.5 mg kg^-1^.

Recent studies have shown that fish rich in Se provides healthy nutrients while minimizing risks associated with Hg exposure. Therefore, in addition to total Hg measurement, the Hg health risk associated with consumption of tilapia from U.S. marketplace was investigated by measuring HBV. This criterion considers both Se and Hg concentrations and is proposed as a more reliable risk assessment index in regard to Hg exposures. Tilapia fillets had positive HBV’s which is an indication that health benefits outweigh risks related to Hg exposures.

Mineral contribution to DRI per 100 gram serving for essential and trace minerals calculated. Food that provide more than 10% of a nutrient per serving are considered good source of that nutrient [48]. Tilapia fillets from the U.S. marketplace of this study can provide more than 10% of the select minerals including Se, P for adult and Cu, Mg, P, Se for children and hence are considered good source of these minerals.

Comparison of mineral concentrations in tilapia fillets from marketplace to USDA composition data for raw tilapia fillets showed that tilapia fillets from this market survey had different concentrations of Ca, Cu, Fe, Mg, Mn, K, Na and Zn compared to USDA composition data [49] (Table 7). These differences can be explained by factors such as the geographical variations, types of the diet, the environment that fish were raised in and differences in the methods that has been used to measure the minerals [50].

Diets in the fish trial were supplemented with 1 mg kg^-1^ of organic or inorganic Se. Dietary Se level of 2.06 mg kg^-1^ has been shown to be beneficial for Nile tilapia and this requirement cannot be met by an aquafeed that is not enriched with Se [51]. During the 7-week fish trial fish demonstrated expected performance. Survival was 100% for all the treatment groups with a mean weekly weight gain of 41 and 45 grams for inorganic and organic Se fed fish, respectively.

There were no significant differences in the FCR, weight gain, HSI, VSI, hepatic and plasma GPx activity, or in the intestinal microvilli length of fish from the different treatment groups. Similar results were observed in studies on Crucian carp (*Carassius auratus gibelio*), juvenile grouper (*Epinephelus malabaricus)* and rainbow trout (*Oncorhynchus mykiss)* [52–55]. However, as we hypothesized, feeding the organic form of Se led to a significant increase in Se concentrations observed in kidney, plasma and fillets of the fish. This appears to be due to differences in the absorption, bioaccumulation and bioavailability of different species of Se (organic and inorganic) [56]. Selenomethionine (SeMet), an organic form of Se that was used in this study, is readily absorbed by erythrocytes through the same active mechanism that incorporates methionine (Met), the more abundant sulfur analogue. Since Met and SeMet are incorporated into cellular proteins for repeated protein synthesis cycles until they are eventually degraded several weeks to several months later, organic Se fed in this form tends to be retained for extended periods of time. In contrast, sodium selenite, an inorganic form of Se, is digestively absorbed and rapidly incorporated into Sec. Since Sec cannot be reused in protein synthesis but must instead be actively degraded into inorganic metabolites which are readily excreted when sufficient Se is available, dietary Se provided in the inorganic form is retained to lesser extents in the muscles and is excreted in higher concentrations than organic Se. Because Met and SeMet are immediately incorporated into tissues with high levels of protein synthesis such as skeletal muscles, liver, and kidney, these tissues accumulate greater amounts of Se [57]. Since no significant differences in GPX activity were noted between the two treatment groups, this confirms that the diets were Se-replete.

Comparison of the concentrations of Se in the fillets of market samples and fillets from the fish trial showed that fillets from fish fed organic Se had significantly higher(*p<0*.*05*). Se contents. To maintain normal physiological functions, the DRI for adults consume 0.53 μg of Se per day and children (4–8 y) consume 30 μg of Se per day. A 100 g serving of tilapia fillets enriched with organic Se in this study had 0.56 μg of Se which satisfies the recommended intake level per day completely for both children and adult. However, fillets from market study and organic fed fish had 0.22 and 0.2 μg of Se per serving (100 g) that can only satisfy 68% of DRI for children and 38% of DRI for adults.

Thus, this study highlights the importance of production and promoting the consumption of value -added fish fillets for combating micronutrient deficiency. While undernutrition can be improved by increasing energy intake, micronutrient deficiency is of a different nature and result from inadequate quality of diet.

In poorer households and developing countries there are limited affordability, availability and even cultural acceptability of food from animal sources that are main sources of micronutrients. In this respect fish offer an advantage as they are more acceptable, more available and consumed by preference in various regions of the developing world [58]. Therefore, promoting the consumption of value-added fish fillets (i.e. Se enriched) can be used as a whole food approach for solving the problem such as Se deficiency and heavy metal toxicity in developing countries [59].

In conclusion, this study demonstrated that organic Se is a more bioavailable form of Se in Nile tilapia. To produce Se enriched tilapia fillets, organic species of Se should be used by the aquaculture industry as an alternative to inorganic species.
